# Epidemiological investigation of non*-albicans Candida* species recovered from mycotic mastitis of cows in Yinchuan, Ningxia of China

**DOI:** 10.1186/s12917-018-1564-3

**Published:** 2018-08-29

**Authors:** Jun Du, Xiaoyu Wang, Huixia Luo, Yujiong Wang, Xiaoming Liu, Xuezhang Zhou

**Affiliations:** 1 Key Laboratory of the Ministry of Education for the Conservation and Utilization of Special Biological Resources of Western China, Ningxia University, Yinchuan, 750021 Ningxia China; 2 College of Life science, Ningxia University, Yinchuan, 750021 Ningxia China

**Keywords:** Mycotic mastitis, *Candida parapsilosis*, *Candida krusei*, Antimicrobial susceptibility, Virulent gene

## Abstract

**Background:**

*Candida spp.* is the vital pathogen involved in mycotic mastitis of cows. However the epidemiology and infection of *Candida* species in mycotic mastitis of cow in Ningxia province of China has not been explored. In the present study, the epidemiology, antimicrobial susceptibility and virulence-related genes of non-albicans Candida (NAC) species were investigated.

**Methods:**

A total of 482 milk samples from cows with clinical mastitis in four herds of Yinchuan, Ningxia were collected and used for the isolation and identification of mastic pathogens by phenotypic and molecular characteristics, and matrix-assisted laser desorption ionization-time of flight mass spectrometry. The antimicrobial susceptibility to antifungal agents was also determined by a disk diffusion assay. The presence of virulence-related genes was determined by polymerase chain reaction (PCR).

**Results:**

A total of 60 isolates from nine different *Candida* species were identified from 256 (60/256, 23.44%) milk samples. The most frequently identified species in cows with clinical mastitis groups were *Candida krusei* (*n* = 14) and *Candida parapsilosis* (*n* = 6)*.* Others include *Candida lipolytica*, *Candida lusitaniae*, *Cryptococcus neoformans.* But *no Candida albicans* was identified in this study. Interestingly, All *C. krusei* isolates (14/14) were resistant to fluconazole, fluorocytosine, itraconazole and ketoconazole, 2 out of 14 *C. krusei* were resistant to amphotericin, and 8 out of the 14 were resistant to nystatin. Similarly, all six *C. parapsilosis* isolates were resistant to fluorocytosine, but susceptible to fluconazole, ketoconazole and nystatin; two of the six were resistant amphotericin and itraconazole. Molecularly, all of the *C. parapsilosis* isolates carried eight virulence-related genes, FKS1, FKS2, FKS3, SAP1, SAP2, CDR1, ERG11 and MDR1. All of the *C. krusei* isolates contained three virulence-related genes, ERG11, ABC2 and FKS1.

**Conclusion:**

These data suggested that *Candida* species other than *C. albicans* played a pathogenic role in mycotic mastitis of cows in Yinchuan, Ningxia of China. The high incidence of drug-resistant genes in *C. parapsilosis* and *C. krusei* also highlighted a great concern in public and animal health in this region.

## Background

Cow mastitis is a disease caused by infections of a variety of microorganisms, which causes large economical looses and damages to the breeding industry by decreasing milk productivity and increasing costs of antibiotic treatments and culling [1]. In most cases, fungal infections of the mammary gland are mostly caused by yeast, the main genus of which is Candida [2]. Mastitis infections caused by fungi of the *Candida* genus have long been known in animals, and *Candida-*caused mycotic mastitis was firstly described by Fleischer in 1930 [3]. In this regard, *Candida* species are considered as opportunistic pathogens that colonize the cow udder. In addition, the harness and abuse of antibacterial agents, and treatments of contaminated antibiotic solutions, as well as duct, or other materials brought in contact with the mammary gland also favor yeast colonization in cow udders [4].

Recently, a large number of virulent factors have been found in *C. parapsilosis* and *C. krusei* originated from cow mastitis, including the Fksp subunits of the β-1,3-D-glucan synthase enzyme (FKS1, FKS2, and FKS3), aspartyl proteinases (SAPP1 and SAPP2), the ATP binding cassette (ABC) transporters, *Candida* drug resistance gene 1 (CDR1), major facilitator superfamily (MFS) transporter multiple drug resistance gene 1 (MDR1), 14α-demethylases (ERG11), efflux pump transporters (ABC1 and ABC2). These factors favor the survival and growth of *Candida spp.* in the mammary gland of cows.

The dairy industry is a predominant economics in Ningxia, a province in Western China. The mastitis of cows causes a significant economic loss in this region every year. It has been well documented that mycotic mastitis is an important causation of economic loss to the dairy industry, however the epidemiology of pathogens may vary in different areas. In this respect, there is a paucity of information about antimicrobial resistance and virulent factors of *C*. *parapsilosis* and *C. krusei* isolates in Ningxia of China, the aim of the present study is therefore to interrogate the epidemiology of fungal infection, and the antimicrobial susceptibility and virulence-related genes of *Candida spp.* in Yinchuan, Ningxia of China.

## Methods

### Isolation and identification of pathogens

This study was submitted to and approved by the Ethics Committee of Animal Study in Ningxia University. A total of 482 milk samples were collected from the cows with clinical mastitis, which originated from four herds in Yinchuan of Ningxia province, China. Clinical mastitis was defined by swelling, reduced milk flow, and abnormal milk appearance (watery to viscous with clots varying from gray-white to yellowish). Additionally, other signs of infection such as fever, inappetence, ataxia, and depression were also considered. The samples were first plated onto the Sabouraud dextrose broth and incubated at 37 °C for 48 h. The cultures were identified by morphological characteristics (formation of chlamydoconidium, pseudohyphae and germinal tube development), Gram staining, biochemical tests (growth in the presence of 0.1% cyclohexamide (SigmaTM), acidic pH tolerance, urea hydrolysis and carbohydrates assimilation and/or fermentation) and CHROMagar Candida culture [5]. All presumptive *Candida* species isolates were further confirmed by Matrix- assisted laser desorption ionization-time of flight mass spectrometry (MALDI-TOF MS) (VITEK® MS, BioMerieux, France).

### Antimicrobial susceptibility tests

The antimicrobial susceptibility was determined by disk diffusion method according to the guideline of the Clinical Laboratory Standards Institute for antifungal susceptibility [6, 7]. A total of six antimicrobial agents were used to evaluate the antimicrobial resistance of the isolates, which included fluorocytosine (1 μg/disk), itraconazole (10 μg/disk), ketoconazole (15 μg/disk), fluconazole (25 μg/disk), nystatin (50 μg/disk), amphotericin (10 μg/disk). Results were recorded as resistant, intermediate and sensitive. The *C. parapsilosis* ATCC 22019 strain and *C. krusei* ATCC 6258 strain were used as references for *C. parapsilosis* strain and *C. krusei*, respectively.

### Detection of virulence-related genes

The presence of virulence-related genes in C. *parapsilosis* and *C. krusei* isolates was detected by a PCR assay. The genes of interest, primer sequences, and expected size of fragment of PCR products were given in Tables 1 and 2. The PCR reactions were performed in a final volume of 25 μL of reaction mixture consisted of 50 ng of genomic DNA, 20 pmol of each primer, and 12.5 μL of 2× Taq PCR MasterMix containing 0.1 U of Taq polymerase/μL, 0.5 mM dNTP each, 20 mM Tris-HCl/pH 8.3, 100 mM KCl, 3 mM MgCl_2_ (Tiangen Biotech, Beijing, China). The cycling conditions were as following: an initial denaturation at 94 °C for 3 min; 30 cycles of denaturation at 94 °C for 30 s, annealing at distinct temperature (Tables 1 and 2) for 30 s, and primer extension at 72 °C for 1 min; and a final extension at 72 °C for 6 min.

**Table 1:** The PCR primers for amplification of *C. parapsilosis* virulence genes

Gene	Primer sequence^a^ (5′–3′)^a^	Annealing temperature	Reference
FKS1	F: ATCCAAGATCTTCCGGTGCCTCAA	60°C	[8]
	R: ATCAGCTGACCATGCTGGATATGG		
FKS2	F: AATGGGCAGAGGTTGAGAAGGTAG	60°C	
	R: GGGTTCCAAGCAGGATATGGATCA		
FKS3	F: TCGTAGGTTCGAATCCTGCTGAGA	60°C	
	R: ATGGTGAAGGCGCAACGGTGTAAA		
SAPP1	F: ACTGGACAACAAATTGCAGATG	57°C	[9]
	R: TAAACTGCTTCATTGCTGGTGT		
SAPP2	F: GTCATATGGGGGATTTGCAC	57°C	
	R: CGCTTTGCTGATGTTACCAG		
MDR1	F: TTCGTGATAGTTTTGGTGGTAG	62°C	[10]
	R: TGAACCTGGAGTGAATCTTGT		
CDR1	F: ATTTGCCGACATCCACCGTTAGG	60°C	[11]
	R: ACCATGCTGTTTGCGAGTCCA		
ERG11	F: GTACACCGTCATTACTCTACCCAACA	62°C	
	R: TGCTCCTTTCATTTACAACATCATTT		

^a^*F* forward, *R* reverse

**Table 2:** The PCR primers for amplification of *C. krusei* virulence genes

Gene	Primer sequence^a^ (5′–3′)^a^	Annealing temperature	Reference
FKS1	F: ACTGCATCGTTTGCTCCTCT	63°C	[12]
	R: GAACATGATCAATTGCCAAC		
ABC1	F: GATAACCATTTCCCACATTTGAGT	60°C	[13]
	R: CATATGTTGCCATGTACACTTCTG		
ABC2	F: CCTTTTGTTCAGTGCCAGATTG	60°C	
	R: GTAACCAGGGACACCAGCAA		
ERG11	F: ATTGCGGCCGATGTCCAGAGGTAT	60°C	
	R: GCGCAGAGTATAAGAAAGGAATGGA		

^a^*F* forward, *R* reverse

## Results

### Isolation and identification of COW mastitis pathogens

Total of 256 pathogenic yeasts were isolated from 482 milk samples collected from cows with clinical mastitis (256/482, 53.1%) in Yinchuan of Ningxia province. Among them, 60 (60/256, 23.44%) were identified in nine of Candida species, in which the *C. krusei* (23.33%, 14/60) and *C. parapsilosis* 10% (6/60) were two of the most frequent Candida species. In addition, 16.66% isolates (10/60) were classified as *Candida*-like species. Other identified Candida species included *Candida lipolytica* (5/60, 8.33%)*, Candida lusitaniae* (5/60, 8.33%), *Candida rugosa* (4/60, 6.67%)*, Trichosporon mucoides* (4/60, 6.67%)*, Candida sphaerica* (3/60, 5%)*, Candida tropicalis* (3/60, 5%) and *Candida utilis* (3/60, 5%).

**Fig. 1:**
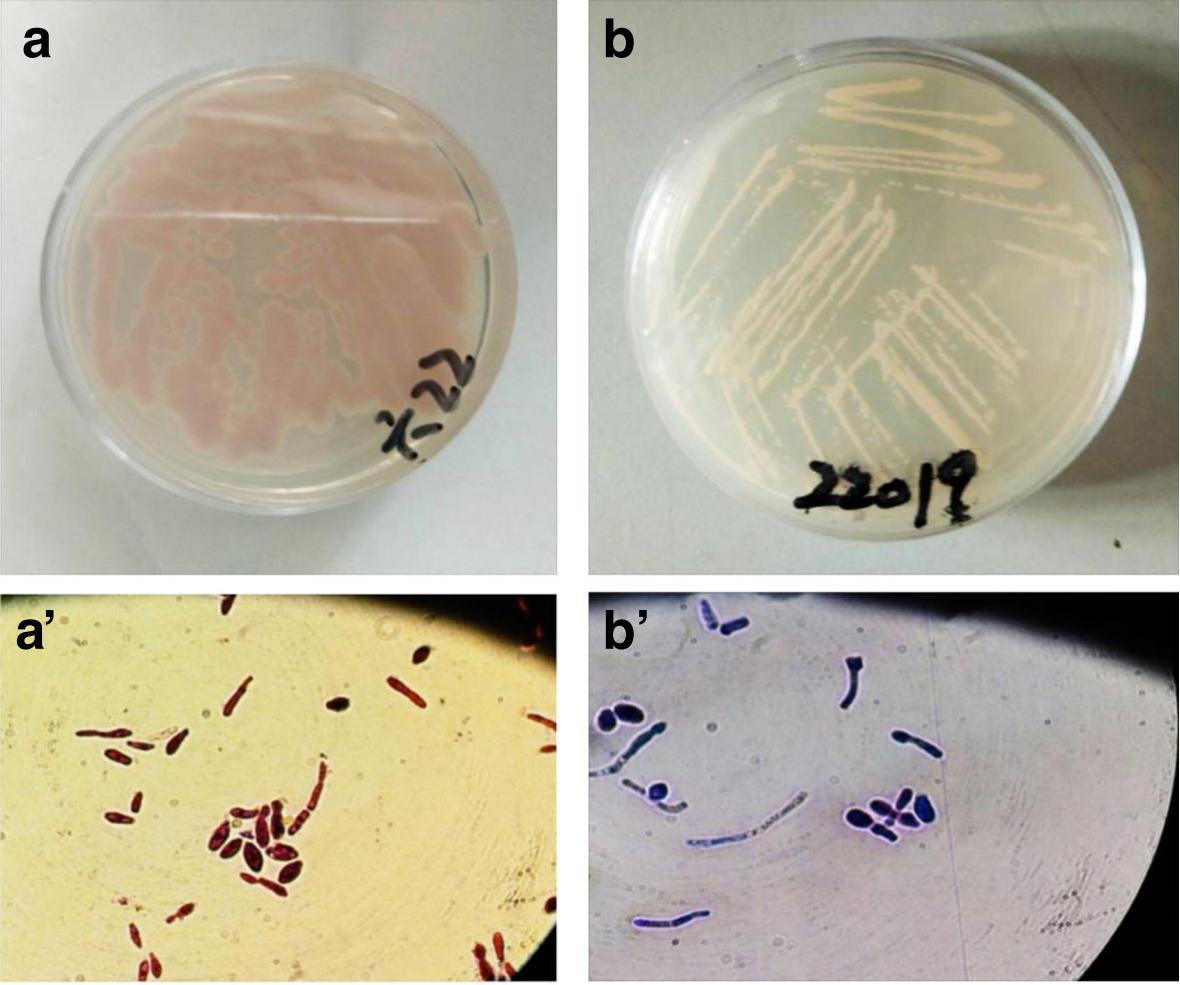
Representative culture and Morphology of *C. krusei* and *C. parapsilosis* isolates. **a** Representative culture of *C. krusei* on CHROMagar plate; **a’** the morphology of Giemsa staining for yeast and pseudohyphae of a *C. krusei* isolate in **a**; **b** Representative culture of *C. parapsilosis* on CHROMagar plate; **b’** the morphology of Giemsa staining for yeast and pseudohyphae of a *C. parapsilosis* isolate in (**b**)

CHROMagar Candida is a differential culture medium that allows selective isolation of yeasts and simultaneously identifies colonies of *C. albicans*, *C*. *krusei*, and other *Candida* spp. Results of this study confirm the accuracy of CHROMagar in providing a presumptive identification of *C*. *krusei* and *C. parapsilosis*, which was consistent with Odds and Bernaerts study [8]. These results of the preliminary identification on the CHROMagar Candida were agree with those by MALDI-TOF MS. *C. krusei* is the only species which grows on Sabouraud’s dextrose agar as spreading colonies with a matt or a rough whitish yellow surface, in contrast to the convex colonies of other Candida spp. All 14 isolates of *C. krusei* tested formed colonies after 48 h of incubation at 37 °C on CHROMagar Candida that were typically pale pink, large, rough, flat, and spreading with broad white edges (Fig. 1a). Unlike *C. krusei*, all 6 isolates of *C. parapsilosis* formed white or faint yellow, small, papillae, smooth colony (Fig. 1b). In addition, *C. krusei* and *C. parapsilosis* are both usually found in two basic morphological forms, as yeast and pseudohyphae (Fig. 1a’ and b’). Both were frequently present simultaneously in growing cultures and may not be separated easily.

### Antimicrobial susceptibility test

**Fig. 2:**
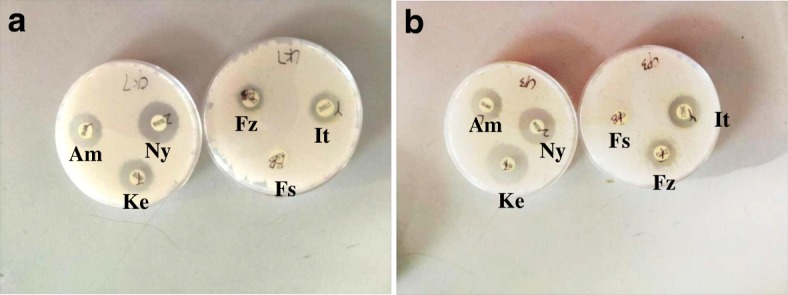
Representative antimicrobial susceptibility tests by disk diffusion assay for *C. krusei* and *C. parapsilosis* isolates. **a** Representative disk diffusion assay for *C. krusei* isolate cultured on CHROMagar plate with indicated antimicrobial disks; **b** Representative disk diffusion assay for a *C. parapsilosis* isolate cultured on CHROMagar plate with indicated antimicrobial disks. Am, amphotericin; Fs, fluorocytosine; Fz, fluconazole; It, itraconazole; Ke, ketoconazole; Ny, nystatin

The antimicrobial susceptibility test demonstrated that *C*. *krusei* and *C. parapsilosis* isolates from cow mastitis cases in Yinchuan of Ningxia province had a variable degree of resistance to the antimicrobials (Fig. 2a and b, Tables 3 and 4). *C. krusei* isolates were respectively resistant to fluconazole (14/14, 100%), fluorocytosine (14/14, 100%), itraconazole (14/14, 100%), ketoconazole (14/14, 100%), amphotericin (2/14, 14.3%), and nystatin (8/14, 57.1%) (Table 3). *C. parapsilosis* isolates were respectively resistant to fluorocytosine (6/6, 100%), amphotericin (2/6, 33.3%), itraconazole (2/6, 33.3%), but susceptible to fluconazole (6/6, 100%), ketoconazole (6/6, 100%), nystatin (6/6, 100%) (Table 4).

**Table 3:** Results of antimicrobial susceptibility tests of *C. krusei* isolates (*n* = 14)

Antibiotic	Resistant, % (no.)	Intermediate, % (no.)	Susceptible-dose dependent, SDD, % (no.)	Susceptible, % (no.)
Amphotericin	14.3 (*n* = 2)	85.7 (*n* = 12)	0	0
Fluorocytosine	100 (*n* = 14)	0	0	0
Fluconazole	100 (*n* = 14)	0	0	0
Itraconazole	100 (*n* = 14)	0	0	0
Ketoconazole	100 (*n* = 14)	0	0	0
Nystatin	0	42.9 (*n* = 6)	0	57.1 (*n* = 8)

**Table 4:** Results of antimicrobial susceptibility tests of *C. parapsilosis* isolates (*n* = 6)

Antibiotic	Resistant, % (no.)	Intermediate, % (no.)	Susceptible-dose dependent, SDD, % (no.)	Susceptible, % (no.)
Amphotericin	33.3 (*n* = 2)	0	33.3 (*n* = 2)	33.3 (*n* = 2)
Fluorocytosine	100 (*n* = 6)	0	0	0
Fluconazole	0	0	0	100 (*n* = 6)
Itraconazole	33.3 (*n* = 2)	0	66.7 (*n* = 4)	0
Ketoconazole	0	0	0	100 (*n* = 6)
Nystatin	0	0	0	100 (*n* = 6)

#### Detection of Virulence-related Genes

**Fig. 3:**
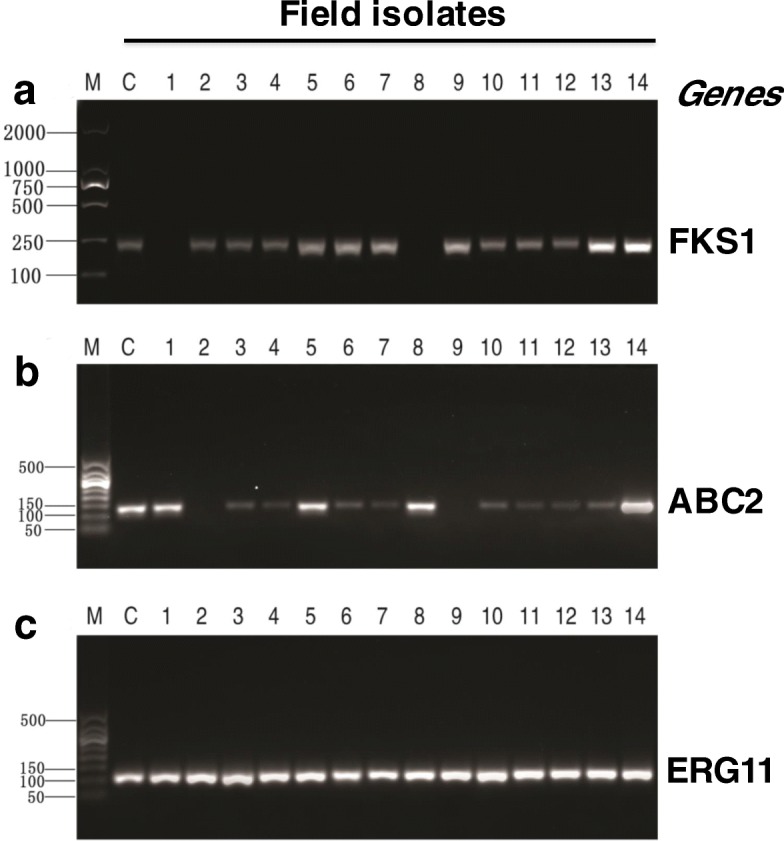
Virulence-related genes of *C. krusei* isolates determined by PCR assay. The indicated virulence-related genes of (**a**) FSK1, **b** ABC2 and (**c**) EGR11 in 14 field *C. krusei* isolates of this report were detected by PCR assay. C, control *C. krusei* ATCC6258 strain; lanes 1 to 14 represented *C. krusei* isolate 1–14. M, 50 bp DNA ladders

**Fig. 4:**
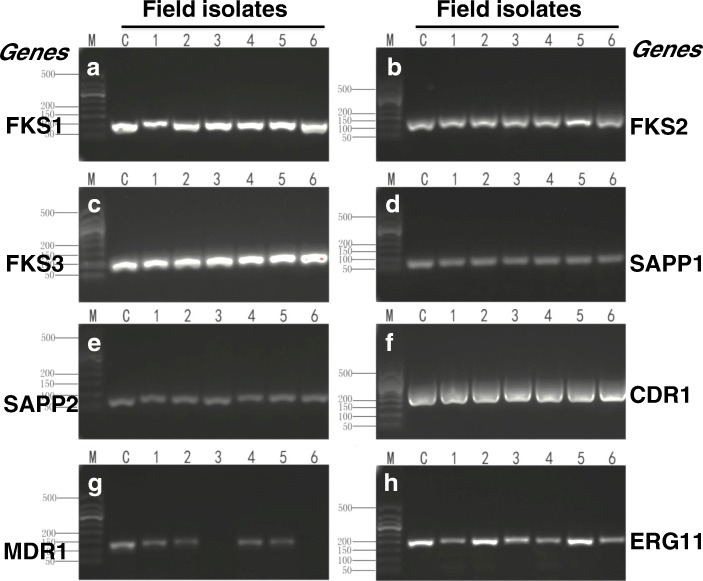
Virulence-related genes of *C. parapsilosis* isolates determined by PCR assay. The indicated virulence-related genes of (**a**) FSK1, **b** FSK2, **c** FSK3 (**d**) SAPP1, **e** SAPP2, **f** CDR1, **g** MDR1 and (**h**) EGR11 in 6 field *C. parapsilosis* isolates of this report were detected by PCR assay. **c**, *C. parapsilosis* ATCC22019 control strain; lanes 1 to 6 represented *C. parapsilosis* isolate 1–6. M, 2 kb DNA ladders

Three virulence-related genes in *C*. *krusei* and eight virulence-related genes in *C. parapsilosis* isolates were determined by PCR in the present study. The results indicated that all (14/14, 100%) *C. krusei* isolates contained the ERG11 gene, 12 out of 14 (85.7%) *C. krusei* isolates were positive in ABC2 and FKS1 genes, but no ABC1 gene was detected in these *C. krusei* isolates (Fig. 3). All (6/6, 100%) *C. parapsilosis* isolates carried the FKS1, FKS2, FKS3, CDR1, ERG11, SAPP1 and SAPP2 genes; 4 out of the 6 (66.7%) of *C. parapsilosis* isolates contained MDR1 gene (Fig. 4).

## Discussion

In this study, Candida species of yeasts were isolated in the 23.44% (60/256) of all samples analyzed. This rate is a marginally higher than the rates reported in Turkey were 12.7% [9], 17.7% [10]; the rates reported in Brazil were 17.3% [4], 12.8% [11]; and the rate reported in Slovenia was 7.5% [12]. *C. krusei* was the predominant species isolated in this study, which was 23.33% (14/60) of the Candida isolates from those with clinical mastitis. However, there is some discrepancy among rates that were also reported by others. Pengov et al. reported a 34% [12], De&Marin et al. reported *a* 44.5% [4], and Sartori et al. reported a 34.6% [11] of *C. krusei* isolates from cows with mastitis. Their data reported a higher frequency of *C. krusei* infection than findings in this report. However, the rate of *C. krusei* isolates of our finding was higher than others. Ruz-Peres et al. reported a 18.18% [13], ErbaŞ et al. reported a 17.4% [10], Ksouri et al. reported a 15.57% [14], Krukowski et al. reported a 15.5% [15], and Eldesouky et al. reported a 12.2% of *C. krusei* isolates in cow mastitis [16].

*C. parapsilosis* was the second most frequent *Candida* species in this study. Although often being considered less virulent than *C. albicans*, *C. parapsilosis* is the Candida species with the largest increase in clinical incidence in human beings in recent decades. In animals, *C. parapsilosis* are also of great importance in veterinary medicine because of their ability to infect many animal species, including cows [14, 17, 18]. Indeed, many studies have described the isolation of *C. parapsilosis* from milk samples with a frequency varied from 1.7% to up to 25.4%, depending on the sanitary condition and environmental factors [10, 11, 15, 19].

The discrepancy in determination rates of *Candida* species among different geographic regions might be a consequence effect of several factors, such as the abuse of intramammary antibiotic treatment or the use of dairy farm made antibiotic infusions for mastitis treatments [15], yeast contaminated food or environment [20], inadequate sanitary practices of milking procedures, and the natural resistance to fluconazole of *C. krusei* [21, 22]. The relative cell-surface hydrophobicity (CSH) of *C. parapsilosis*, adherence to host tissues and plastic surfaces such as milkers or other prosthetic materials [23–25], and the presence of pathogenic strains of *C. krusei* might also contribute the high rate of *C*. *krusei* and *C. parapsilosis* in milk samples of cow mastitis [26].

Of note, there was no *C. albicans* was identified in this study. This result was in accordance with the report by ErbaŞ et al. [10]. Although *C. albicans* was considered to be the most frequent cause of fungemia, a number of reports have documented infections caused by *C. parapsilosis, C. krusei* and other NAC species [27], suggesting that the pathogenic role of opportunistic NAC species in this disease. Therefore, it is a necessity to pay more attention in NAC species in order to establish the possible role in mycotic mastitis in the region of Yinchuan, Ningxia of China.

In this study, the antifungal resistance was also investigated. Notably, all of the six *C. parapsilosis* isolates (100%) were resistant to fluorocytosine, and all 14 of *C. krusei* isolates (100%) were resistant to ketoconazole, fluconazole, itraconazole and fluorocytosine. This finding was consistent with a study by Sonmez et al. [28], but was different from reports by others [29–31]. Of interest, these agents are the primary agents against the infection of *Candida* species. It has been reported that the resistance of *C. albicans* to fluorocytosine was up to 10–30%, and the incidence of drug resistant NAC species was increased [10, 28]. For example, the natural or acquired resistance to fluconazole was confirmed in *C*. *krusei* and *C. glabrata* [32]. Our finding in *C. krusei* also supported the results reported in literatures. It was worthy to note that *C*. *krusei* and *C. parapsilosis* isolates from cow mastitis cases in Ningxia region were more resistant to antifungal agents. It is strongly recommended that fluorocytosine, and other azole antifungal agents should not be used in the treatment with NAC species in dairy cows in Ningxia province of China, owing to the high drug resistance of these *Candida* species. Interestingly, these isolates were from animals that had not been treated with antifungal agents, suggesting that animal isolates were more exposed to anthropic environmental selective pressures, such as the use of azole antifungals in agricultural practices [33]. Therefore, a special attention must be paid to dairy products for monitoring the antifungal susceptibility of Candida spp. and other fungi recovered from animals is extremely important, since they may act as reservoirs of strains causing human disease and may present a risk for immunocompromised patients [34].

Previous reports indicated that a large number of virulent factors have been determined in *C*. *krusei* and *C. parapsilosis,* including the β-1,3-D-glucan synthase enzyme, aspartyl proteinases, the ATP binding cassette (ABC) transporter, major facilitator superfamily (MFS) transporter, the zinc cluster transcription factor,14α-demethylase, efflux transporters. In the present study, we also observed that most of the *C. krusei* isolates harbored ERG11, ABC2 and FKS1 genes, and most of the *C. parapsilosis* isolates harbored genes of FKS1, FKS2, FKS3, SAPP1, SAPP2, ERG11, CDR1 and MDR1. The Fksp subunits of the β-1,3-D-glucan synthase enzyme are the target of echinocandins [35] and are encoded by the FKS1,FKS2, and FKS3 genes. Many reports indicated that a reduced-susceptibility (RES) to echinocandin was associated with mutations in two conserved regions of fks1 and fks2, and amino acid substitutions in the proteins encoded by these genes were observed within two or three hot spot (HS) regions on each gene [36–38]. In the present study, FKS1, FKS2, and FKS3 genes were detected in all *C. parapsilosis* field isolates. 85.7% (12/14) of *C. krusei* isolates were positive in FKS1 gene. Further studies are needed to confirm the mutation in hot spot (HS) regions on FKS1, FKS2 genes of *C. krusei* and *C. parapsilosis*, in order to elucidate the capability of the M27-A3 guidelines to detect the resistance to echinocandin. The secretion of aspartyl proteinases (Saps) has been considered as important elements of virulence for *C. albicans* [39], and it is also recognized as virulence factors for *C. parapsilosis* [40]. In *C. parapsilosis*, SAPP1, and SAPP2 are two annotated secreted aspartyl proteinase genes [41–43]. These enzymes are involved in providing nutrients for pathogen propagation, tissue colonization and further tissue invasion by rupturing host mucosal membranes [23]. In this study, 100% of *C. parapsilosis* isolates were found to carry SAPP1 and SAPP2 genes. This result was in agreement with previous reports [44].

The common mechanisms of *Candida* resistant to azoles include changes in target enzyme and upregulation of multidrug resistance protein (MDR). The target enzyme of azoles is 14α-lanosterol demethylase (14-DM), which is responsible for the production of an ergosterol precursor and is encoded by the gene *ERG11*. In *C*. *albicans* and *C. parapsilosis*, the efflux pump genes associated with azole resistance include CDR1, CDR2, and MDR1 [45]. However, the drug-resistant genes in *C. krusei* are involved in ABC1 and ABC2 [46, 47]. The results in this study also showed that 100% of *C. parapsilosis* isolates contained the *CDR1* and *ERG11* genes, and 66.7% of *C. parapsilosis* isolates contained the *MDR1* gene. 100% (14/14) of *C. krusei* isolates contained the *ERG11* gene, and 85.7% (12/14) of *C. krusei* isolates contained the *ABC2* gene, despite the *ABC1* gene was not detect in *C. krusei.* This finding was in line with previous studies in humans by He et al. that ABC2p was suggested to play a more important role in the resistance of *C. krusei* to azoles, instead of ABC1p [46].

Taken together, it was demonstrated that *C*. *krusei* and *C. parapsilosis* isolates from mycotic mastitis were present in Yinchuan of Ningxia with a potential of multidrug resistance. Further studies are needed to confirm mutations in ERG11, gain-of-function mutations in transcription factors, such as multidrug resistance regulator 1 (*MRR1*) and transcriptional activator of CDR gene 1 (*TAC1*), and the overexpression of these genes to elucidate the molecular mechanisms of resistance which caused by *C. krusei* and *C. parapsilosis* isolates from mycotic mastitis presented in Yinchuan, Ningxia of China.

## Conclusions

A total of 60 isolates obtained from clinical mastitis milk samples in Yinchuan, Ningxia province of China were identified as *Candida* species according to phenotypic characteristics and MALDI-TOF MS. The most frequent *Candida* species found in this study was *C. krusei*, followed by *C. parapsilosis.* Other non-albicans *Candida* (NAC) species were also found in a low frequency. There was no *Candida albicans* was isolated in this study. According to results of this study, NAC may play an important pathogenic role in mycotic mastitis in dairy farms of Yinchuan region in Ningxia province of China. The *C. krusei* and *C. parapsilosis* displayed a strong antifungal resistance with drug-resistant genes. Most of *C. krusei* isolates harbored the ERG11, ABC2 and FKS1 genes, and most of the *C. parapsilosis* isolates harbored the FKS1, FKS2, FKS3, SAPP1, SAPP2, ERG11, CDR1 and MDR1 genes.

## Abbreviations

14-DM: 14α-lanosterol demethylase (14-DM)
ABC: ATP binding cassette
ATCC: American Type Culture Collection;
C.: Candida
CDR1: Candida drug resistance gene 1
CSH: Cell-surface hydrophobicity
ERG11: 14α-demethylases
FKS: Fksp subunits of the β-1,3-D-glucan synthase enzyme
MALDI-TOF MS: Matrix-assisted laser desorption ionization-time of flight mass spectrometry
MDR1: Multiple drug resistance gene 1
MFS: Major facilitator superfamily
MRR1: Multidrug resistance regulator 1
NAC: Non-albicans Candida
PCR: Polymerase chain reaction
SAP: Secreted aspartyl proteinase
ssp.: Species
TAC1: Transcriptional activator of CDR gene 1
